# Letter to the editor: influenza-associated mortality and oseltamivir: beware of misstepping into stepwise procedures

**DOI:** 10.2807/1560-7917.ES.2019.24.46.1900678

**Published:** 2019-11-14

**Authors:** Theodore Lytras, Stefanos Bonovas, Georgios K. Nikolopoulos, Sotirios Tsiodras

**Affiliations:** 1 National Public Health Organization, Athens, Greece; 2 Department of Biomedical Sciences, Humanitas University, Milan, Italy; 3 Humanitas Clinical and Research Center, Milan, Italy; 4 Medical School, University of Cyprus, Nicosia, Cyprus; 5 4th Department of Internal Medicine, Attikon University Hospital, University of Athens Medical School, Athens, Greece

**Keywords:** oseltamivir, influenza, mortality, stepwise regression, bias, confounding

**To the editor:** We read with interest the recent study by Reacher et al. [1] on risk factors for mortality in hospitalised patients with influenza A(H3N2). The authors assert that a standard 5-day course of oseltamivir cut the odds of death for these inpatients down to one third (adjusted odds ratio (aOR): 0.32; 95% confidence interval (CI): 0.11–0.93). Unfortunately, this impressive aOR serves less as a useful result for clinicians, and more as a useful reminder of the pitfalls of stepwise regression and variable selection.

According to Harrell, “*stepwise variable selection is one of the most widely used and abused of all data analysis techniques*” that “*violates every principle of statistical estimation and hypothesis testing*” [2]. Stepwise regressions produce inappropriately narrow CIs (or low p values) that do not take multiple testing into account, and regression coefficients that are biased away from the null [2]; this has been repeatedly shown in the epidemiological literature, especially for models with many predictors and few events [3]. Moreover, stepwise procedures do not adequately control for confounding, ignore the underlying causal structure thereby potentially introducing collider bias, and should not be used for causal inference [4].

In Table 1 of their article, Reacher et al. report an unadjusted OR of 0.86 (95% CI: 0.34–2.18) indicating no association of standard-course oseltamivir with mortality, which falls to a statistically significant 0.32 in a multivariable model derived via a stepwise procedure (presented in the report’s Table 2, despite a p = 0.23 for the overall variable) [1]. Unfortunately, this ‘adjusted’ OR is entirely non-significant if one accounts for the multiple testing, and is most likely explained by the bias associated with the procedure, as well as uncontrolled confounding due to uncritical inclusion of covariates in the model [5]. Although difficult to sort out, the variable ‘acquisition of infection within hospital’ is particularly suspect, as it could conceivably be associated with both exposure and outcome through unobserved variables, resulting in a form of collider bias known as ‘M-bias’ [6].

Further important problems exist in the analysis. The final multivariable model contains nine predictors in a dataset with just 32 outcome events (deaths), far below the minimum 10 events per variable recommended for a logistic regression; thus the model is probably severely overfitted, which can further bias regression coefficients away from the null [7]. In addition, the low prevalence of several risk factors in the data (for example, ‘excessive alcohol use’, which was found in just eight patients) hints at potential multicollinearity problems, both during the stepwise procedure and in the final model. At a minimum, the authors should have calculated and presented Variance Inflation Factors for the covariates included in their final model in Table 2 [1]. Moreover, as the authors admit, patients can die or be discharged before they have the opportunity to receive oseltamivir; therefore immortal time bias is a very real concern, and time-dependent survival analysis should have been used instead of logistic regression [8]. Also in Table 3 of the Reacher et al. article [1], where analysis of the delay between symptom onset and oseltamivir treatment is presented, the ‘n = 299’ in the title suggests the analysis has somehow included many of the 73/332 patients who did *not* receive oseltamivir at all, and we would kindly ask the authors to clarify.

Some additional points in the study merit attention [1]. No difference in the association with mortality was observed between standard-course oseltamivir (75 mg twice daily for 5 days) and non-standard or modified courses (Table 2 of the report). Moreover, according to Table 1, more than half of all patients started oseltamivir later than the recommended 48 hours from symptom onset, in which oseltamivir has demonstrated effectiveness in alleviating influenza symptoms [9]. This makes the study findings even more implausible. Admittedly, it would be comforting to presume that a few doses of oseltamivir several days from symptom onset might lower a patient’s odds of dying by two thirds. But we would think that if something looks too good to be true, then it is most likely due to bias, especially when problematic stepwise procedures are involved.
